# PacBio single-molecule long-read transcriptome sequencing and analysis of somatic embryogenesis in *Picea mongolica*

**DOI:** 10.3389/fpls.2025.1682365

**Published:** 2025-10-08

**Authors:** Jinling Dai, Shengli Zhang, Yu’e Bai

**Affiliations:** College of Forestry, Inner Mongolia Agricultural University, Hohhot, China

**Keywords:** *Picea mongolica*, long-read transcriptome, somatic embryogenesis, transcription factors, *BABY BOOM*

## Abstract

**Introduction:**

*Picea mongolica* is an endangered conifer species endemic to Hunshandak Sandy Land, uniquely adapted to extreme desert conditions. However, it faces critical conservation challenges due to slow regeneration rates, limited seed production, and high susceptibility to pathogens, which collectively threaten its population sustainability and genetic diversity.

**Methods:**

We performed long-read transcriptome sequencing of pooled samples from various somatic embryogenesis stages using PacBio SMRT technology. The obtained transcripts were functionally annotated using the Nr, SwissProt, KEGG, and KOG databases. We conducted comprehensive transcript structure analyses, including identification of alternative splicing, SSR loci, lncRNAs, and transcription factors. Furthermore, we cloned the *PmBBM* gene and analyzed its sequence characteristics. Expression patterns of *PmBBM* and other AP2 transcription factor family members during somatic embryogenesis were profiled.

**Results:**

Our analysis generated 12,232 high-quality transcripts. We identified 83 genes with alternative splicing, 1,006 SSR loci, 35 lncRNAs, and 548 transcription factors from 46 distinct families. The *PmBBM* gene was successfully cloned and characterized. Expression profiling revealed dynamic expression patterns of *PmBBM* and other AP2 family members across different stages of somatic embryogenesis.

**Discussion:**

This study establishes the first reference-quality transcriptome database for *P. mongolica* using PacBio long-read sequencing, providing essential genomic resources for this non-model species. Our findings not only enhance the understanding of molecular mechanisms in somatic embryogenesis but also lay a foundation for future functional genomics research, including gene validation and molecular marker-assisted breeding. These results have significant theoretical and practical implications for the conservation and sustainable utilization of this endangered conifer.

## 1 Introduction

*Picea mongolica*, a rare and endemic evergreen conifer species in China that belongs to the Pinaceae family, is exclusively distributed in the southeastern region of Hunshandake Sandy Land in Inner Mongolia (Zou et al., 2001). Its wood is widely used for papermaking and furniture production, and its bark serves as a source of turpentine. This ecologically and economically valuable species exhibits remarkable adaptations, including sand-burial tolerance, cold resistance, and drought endurance (Zhang et al., 2013). Recognized for its robust protective functions, aesthetic morphology, and high economic value, *P. mongolica* is an important ecological-economic tree species (Habuer et al., 2021). Due to low natural regeneration rates, high seedling mortality, limited seed production, and susceptibility to pest infestations, the population resources of *P. mongolica* have been continuously declining. To address these conservation challenges, biotechnological approaches such as somatic embryogenesis (SE) offer a promising avenue for mass clonal propagation, germplasm preservation, and genetic improvement. However, the SE protocol for *P. mongolica* remains inefficient and unreliable, primarily due to a complete lack of understanding of the molecular mechanisms governing this process. Identifying key genes and regulatory networks controlling SE is therefore a critical prerequisite for overcoming these technical barriers.

Early research on *P. mongolica* primarily focused on seedling cultivation, afforestation techniques, introduction, cultivation, and its physiological and biochemical characteristics (Liu et al., 2020; Zou et al., 2003; Liu et al., 2018; Tang et al., 2016). Recently, the number of molecular biology investigations has increased. Transcriptomic study of zygotic embryos at various developmental stages revealed stage-specific differential gene expression patterns, with the most pronounced differences occurring between the early embryogenesis and embryo maturation phases. Transcription factor families, including MYB, WRKY, WOX, AP2, GATA, and TCP, exhibit distinct expression profiles across various embryonic developmental stages (Yan et al., 2021a). A transcriptome comparison of non-embryogenic and embryogenic calli identified 13,267 differentially expressed genes, demonstrating that phytohormone-related, stress-responsive, and signal transduction genes collectively regulate embryogenic competence in *P. mongolica* (Wang et al., 2023). To address the challenges of root elongation and lateral root formation during somatic embryo germination, single-cell transcriptomics was employed to characterize cell types and specific expression patterns during lateral root development, elucidate cellular evolutionary trajectories, and construct molecular regulatory networks (Wang, 2023).

SE is an effective *in vitro* regeneration system and an ideal receptor for genetic transformation with significant biotechnological potential (Guan et al., 2016). Extensive research has established that plant SE is regulated by complex gene networks controlled by phytohormones, including auxins, abscisic acid, and cytokinins (Elhiti et al., 2013; Wójcik et al., 2020). The *BABY BOOM* (*BBM*) gene, a member of the AP2 subfamily within the AP2/ERF superfamily, plays a crucial role in plant growth regulation and stress responses (Jha and Kumar, 2018). *BBM* was initially isolated from immature pollen grains of *Brassica napus*. Ectopic *BBM* expression not only promotes cell proliferation and morphogenesis in both *B. napus* and *Arabidopsis thaliana*, but also successfully induces SE without exogenous hormone application (Boutilier et al., 2002; Kulinska-Lukaszek et al., 2012). *In Larix kaempferi × L. olgensis*, *BBM* plays a regulatory role in adventitious root development (Li et al., 2014; Wang et al., 2019). In *Zea mays*, *BBM* promoted embryonic regeneration, thereby influencing transformation efficiency (Du et al., 2019; Masters et al., 2020). *In Rosa canina*, *RcBBM* overexpression promotes shoot regeneration in both leaf and root explants (Yang et al., 2014). Accumulating evidence has confirmed *BBM’*s multifaceted functions in SE induction, cell proliferation and regeneration enhancement, genetic transformation efficiency improvement, and apomixis induction, establishing BBM as an embryo-specific gene and a marker of embryogenic competence (Yuan et al., 2024; Yavuz et al., 2020). Given the well-established conserved role of AP2 transcription factors, particularly the BBM, as master regulators of cell pluripotency and SE, and given their prominent representation in our preliminary transcriptome data, we focused our subsequent analysis on this family.

While previous transcriptomic studies on *P. mongolica* SE (Dai et al., 2025) have characterized gene expression across developmental stages, a comprehensive catalog of long-read transcript isoforms, which is crucial for accurate gene annotation, alternative splicing analysis, and lncRNA identification, remains lacking. The primary objective of this study was therefore to establish a reference-quality, long-read transcriptome for *P. mongolica* using PacBio SMRT sequencing. To achieve this, a pooled sample strategy was employed to maximize the diversity of transcripts captured for isoform discovery. Building upon this foundational resource, we further sought to clone and characterize the key SE-related gene *PmBBM* and to analyze its expression pattern, thereby elucidating its regulatory role. Our ultimate goal is to identify genetic targets for optimizing SE protocols, thereby facilitating the conservation and sustainable utilization of this endangered conifer.

## 2 Materials and methods

### 2.1 Plant materials

**Figure 1:**
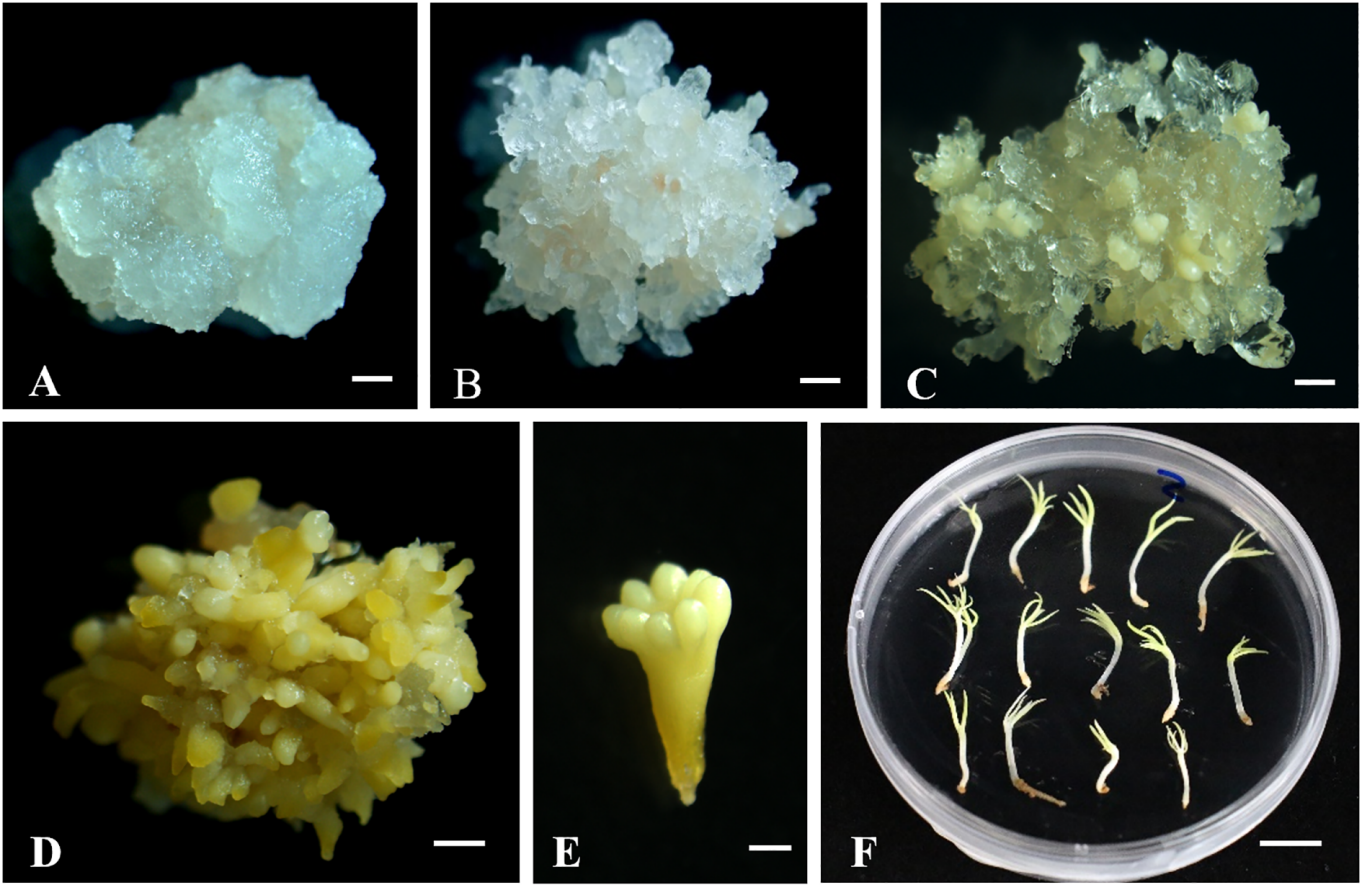
Different stages of *Picea mongolica* somatic embryogenesis. **(A)** Non-embryogenic callus (NEC). **(B)** Embryogenic calli (EC). **(C)** Global Somatic Embryos (GSE). **(D)** Late Somatic Embryos (LSE), **(E)** Mature Somatic Embryos (MSE), **(F)** Somatic Embryo Plantlets (EP). A-E Scale bars = 1 mm. F Scale bars = 1 cm.

The immature seeds of *P. mongolica* were collected from the Baiyinaobao Nature Reserve in Hexigten Banner, Chifeng, Inner Mongolia, China. The SE of *P. mongolica* was induced using immature zygotic embryos as explants, following the established protocol developed by our research group (Yan et al., 2021b). Samples of non-embryogenic callus (NEC), embryogenic callus (EC), global somatic embryos (GSE), late somatic embryos (LSE), mature somatic embryos (MSE), and somatic embryo-derived plantlets (EP) were collected (**Figure 1**). The samples were immediately frozen in liquid nitrogen after collection and subsequently stored at -80 C.

### 2.2 RNA extraction, library construction, and sequencing

Total RNA was extracted by grinding the tissue using a TRIzol reagent (Invitrogen, Carlsbad, CA, USA). RNA integrity was determined using an Agilent 2100 Bioanalyzer (Agilent Technologies, Palo Alto, CA, USA) and agarose gel electrophoresis. The purity and concentration of the RNA were determined with a Nanodrop micro-spectrophotometer (Thermo Fisher Scientific, Waltham, MA, USA).long-read cDNA was synthesized from poly(A)+ RNA using the Clontech SMARTer PCR cDNA Synthesis Kit (Takara, Osaka, Japan), which is specifically designed for PacBio Iso-Seq library preparation. The resulting double-stranded cDNA was PCR-amplified. The SMRTbell template library was constructed following the standard Iso-Seq protocol, which included DNA damage repair, end repair, and adapter ligation. The SMRTbell template was annealed to sequencing primers, bound to polymerase, and sequenced on a PacBio Sequence II platform (Gene Denovo Biotechnology Co., Guangzhou, China).

### 2.3 Data processing

The raw sequencing reads from the cDNA libraries were analyzed using SMRT Link V8.0.0 (Gordon et al., 2015). A high-quality circular consensus sequence (CCS) was extracted from the subread BAM file. Primers, barcodes, poly A tail trimming, and a concatemer of full passes were removed to obtain full-length nonchimeric (FLNC) reads. Similar FLNC reads were used in minimap2 for hierarchical clustering to obtain a consistency sequence. The quiver algorithm was then used to correct the consistency sequence further. Based on the results, high-quality isoforms (prediction accuracy is ≥ 0.99) were used for subsequent analyses (Salmela and Rivals, 2014).

Isoforms were BLAST analyzed against the NCBI non-redundant protein (Nr) database (http://www.ncbi.nlm.nih.gov), Swiss-Prot protein database (http://www.expasy.ch/sprot), Kyoto Encyclopedia of Genes and Genomes (KEGG) database (https://www.genome.jp/kegg/), and Clusters of Orthologous Genes/Eukaryotic Orthologous Groups (COG/KOG) database (http://www.ncbi.nlm.nih.gov/COG) with BLASTx program (http://www.ncbi.nlm.nih.gov/BLAST/). Sequence similarity with genes of other species Gene Ontology (GO) annotation was analyzed using Blast2GO software (Conesa et al., 2005) with the Nr annotation results of the isoforms.

Simple Sequence Repeats (SSR) analysis was performed using MISA, followed by lncRNA identification of long-read transcripts that were not annotated in the four major databases. Coding potential was rigorously assessed using both CNCI (Sun et al., 2013) and CPC (Kong et al., 2007), and only transcripts unanimously predicted as non-coding by both tools were considered reliable lncRNAs. Cogent software was used to assemble the coding sequences, which served as a reference for alternative splicing (AS) analysis using SUPPA (Li et al., 2017; Alamancos et al., 2015). Finally, the predicted protein sequences were subjected to hmmScan against the PlantTFDB database to identify and quantify transcription factors (TFs).

### 2.4 Cloning of the *PmBBM* gene and bioinformatics analysis

Using the long-read transcriptome data, we predicted the CDS of the *PmBBM* gene (Isoform0001869). Gene-specific primers, PmBBM-F (5’-ATGGGGTCGACGAGCAATT-3’) and PmBBM-R (5’-TTATGTGTCGTTCCATACAGTGAAA-3’), were designed for PCR amplification, followed by electrophoresis, gel recovery, T-vector ligation, and transformation into *Escherichia coli* DH5α competent cells. The recombinant plasmids were subsequently sent to Tsingke Biotechnology for sequencing.

The corresponding amino acid sequence was translated using DNAMAN software. The physicochemical properties of the PmBBM protein were analyzed using the online tool ProtParam (https://web.expasy.org/protparam). Transmembrane domains were predicted using DeepTMHMM (https://dtu.biolib.com/DeepTMHMM/) and hydrophobicity analysis was performed using ProtScale (https://web.expasy.org/protscale/). Signal peptide prediction was conducted using SignalP-6.0 (https://services.healthtech.dtu.dk/services/SignalP-6.0/). The secondary and tertiary structures of PmBBM were predicted using SOPMA (https://npsa-prabi.ibcp.fr/cgi-bin/npsa_automat.pl?page=npsa_sopma.html) and SWISS-MODEL (https://swissmodel.expasy.org/), respectively. Phosphorylation sites were predicted using NetPhos (https://services.healthtech.dtu.dk/services/NetPhos-3.1/), and conserved domains were analyzed using NCBI (https://www.ncbi.nlm.nih.gov/Structure/cdd/wrpsb.cgi). The homologous amino acid sequences of PmBBM were obtained from NCBI database (https://www.ncbi.nlm.nih.gov/). DNAMAN was used to the multiple sequence alignments. A phylogenetic tree was built with MEGA6.

### 2.5 Subcellular localization

The overexpression vector pBWA(V)H2STMVΩ-3×flag-BBM was created by connecting *PmBBM* to pBWA(V)H2STMVΩ-3×flag-ccdB-egfp. The overexpression vector was transformed into DH5α receptor cells. Positive clones were identified via colony PCR and sequenced for further identification. The correct plasmid transformed into Agrobacterium GV3101 receptive cells was detected, pBWA(V)H2STMVΩ-3×flag-ccdB-egfp was used as a negative control, positive clones were selected and propagated, Agrobacterium infection solution was prepared, and the OD600 was adjusted to approximately 0.60. The lower epidermis of tobacco leaves was injected with a syringe, cultured in the dark for 48 h, and observed and photographed under a confocal laser microscope (Nikon C2-ER).

### 2.6 Analysis of the expression patterns of the *PmBBM* and AP2 transcription factor family

Utilizing our team’s existing transcriptomic data from NEC, EC, GSE, LSE, MSE, and EP developmental stages, we systematically examined the expression profiles of *PmBBM* and AP2 family genes throughout the six developmental phases. Gene expression patterns were visualized via heatmap analysis using OmicSmart (http://www.omicsmart.com).

## 3 Results

### 3.1 Overview of long-read transcript sequencing data

**Figure 2:**
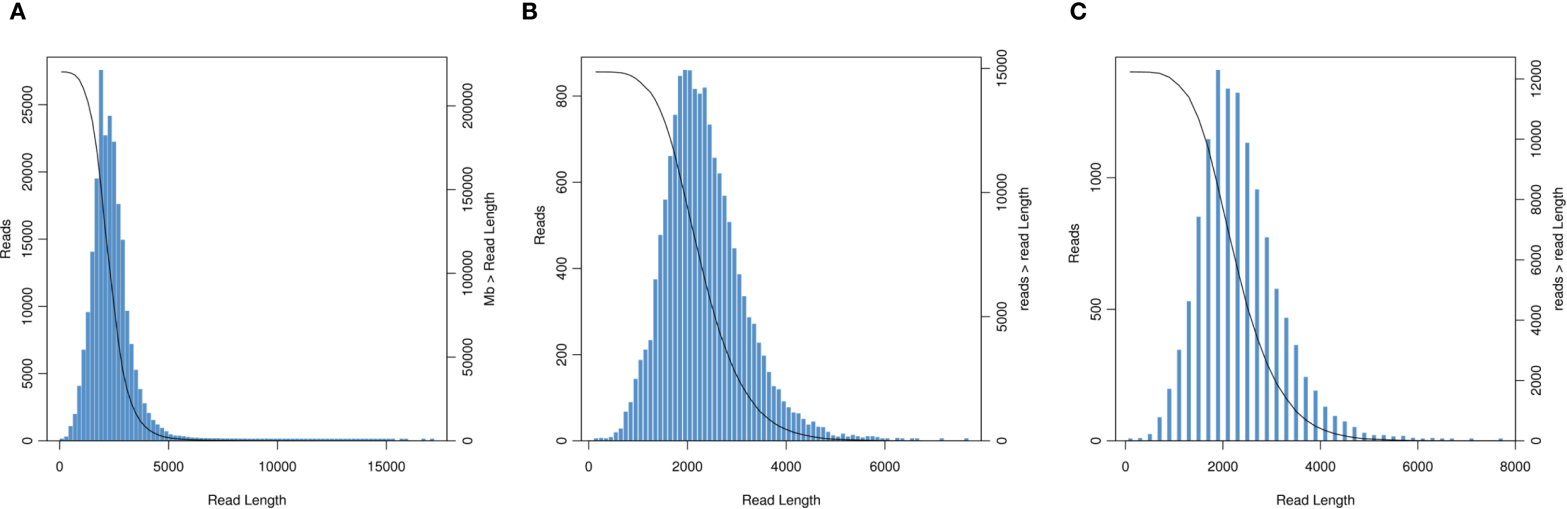
Length distribution of long-read transcripts **(A)** Circular consensus sequence length distribution graph. **(B)** Unpolished consensus isoforms sequence length distribution graph. **(C)** Isoform sequence length distribution graph.

We obtained 33.88 Gb of sequencing data using SMART sequencing, comprising 15,586,833 subreads with an average length of 2,173 bp and an N50 of 2,350 bp (**Figure 2A**). After filtering subreads with Full Passes ≥1, we obtained 220,241 high-accuracy CCS reads totaling 510,826,820 bases, with an average length of 2,319 bp and mean Full Pass number of 42. Classification of these CCS reads yielded 112,449 FLNC sequences (51.06%) averaging 2,320.79 bp, 1441 full-length chimeric sequences (0.65%), and 106,351 non-full-length reads (48.29%) (**Figure 2B**). Hierarchical clustering of the FLNC reads generated consensus sequences that were subsequently polished using the Quiver algorithm to produce 14,823 high-quality and 36 low-quality sequences. After redundancy removal, we obtained 12,232 long-read transcript isoforms with an average length of 2,331.98 bp and an N50 of 2,486 bp, with read lengths predominantly distributed between 1000–4000 bp (**Figure 2C**).

### 3.2 Classification and functional annotation

**Figure 3:**
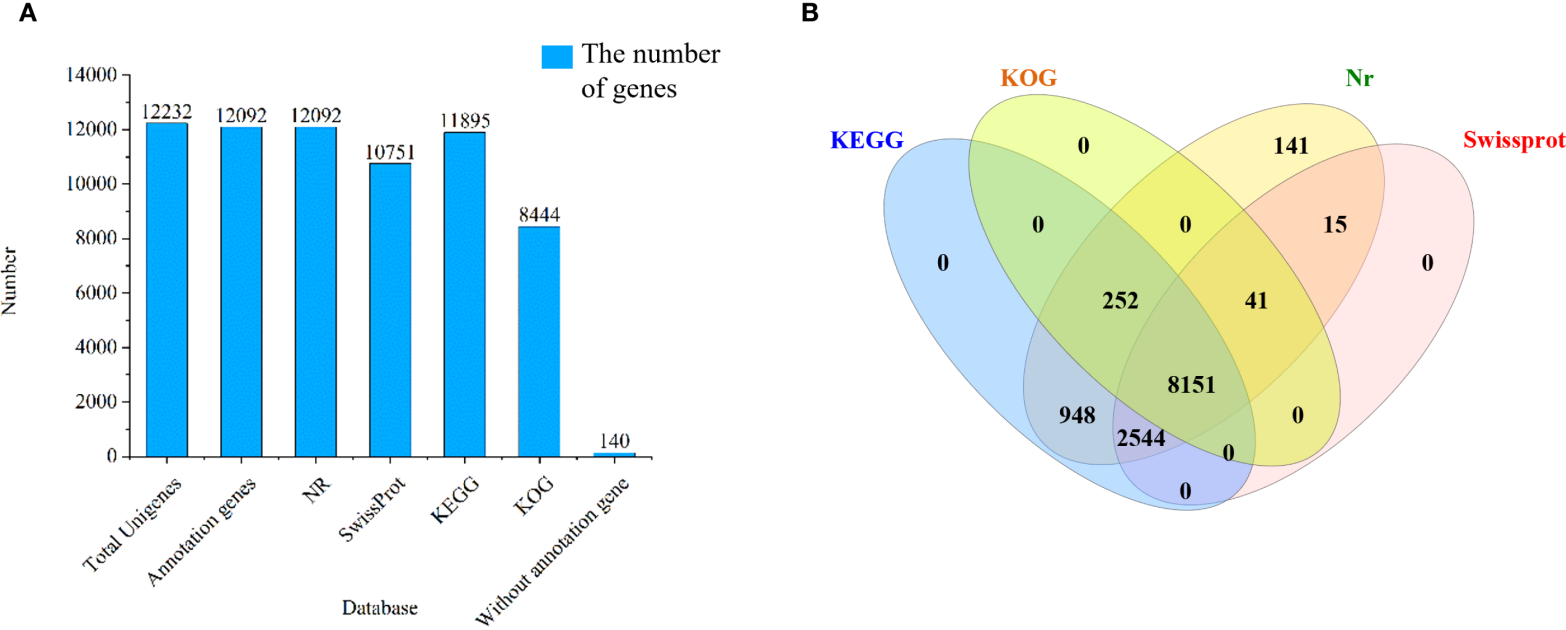
Functional annotation of isoforms in *Picea mongolica.* **(A)** Annotation statistics of long-read transcripts in four datasets **(B)** Four database annotation Venn diagram. KEGG, Kyoto Encyclopedia of Genes and Genomes; KOG, Eukaryotic Orthologous Groups; Nr, Non-redundant protein.

Comparative analysis revealed successful annotation of 12,092 (98.86%) long-read transcripts across KEGG, KOG, Nr, and Swiss-Prot databases. Specifically, 11,895 (97.24%) transcripts were annotated in KEGG, 8,444 (69.03%) in KOG, 12,092 (98.86%) in Nr, and 10,751 (87.89%) in Swiss-Prot, whereas 140 (1.14%) transcripts remained unannotated (**Figure 3A**). Notably, 8,151 transcripts were simultaneously annotated in all four databases (**Figure 3B**).

**Figure 4:**
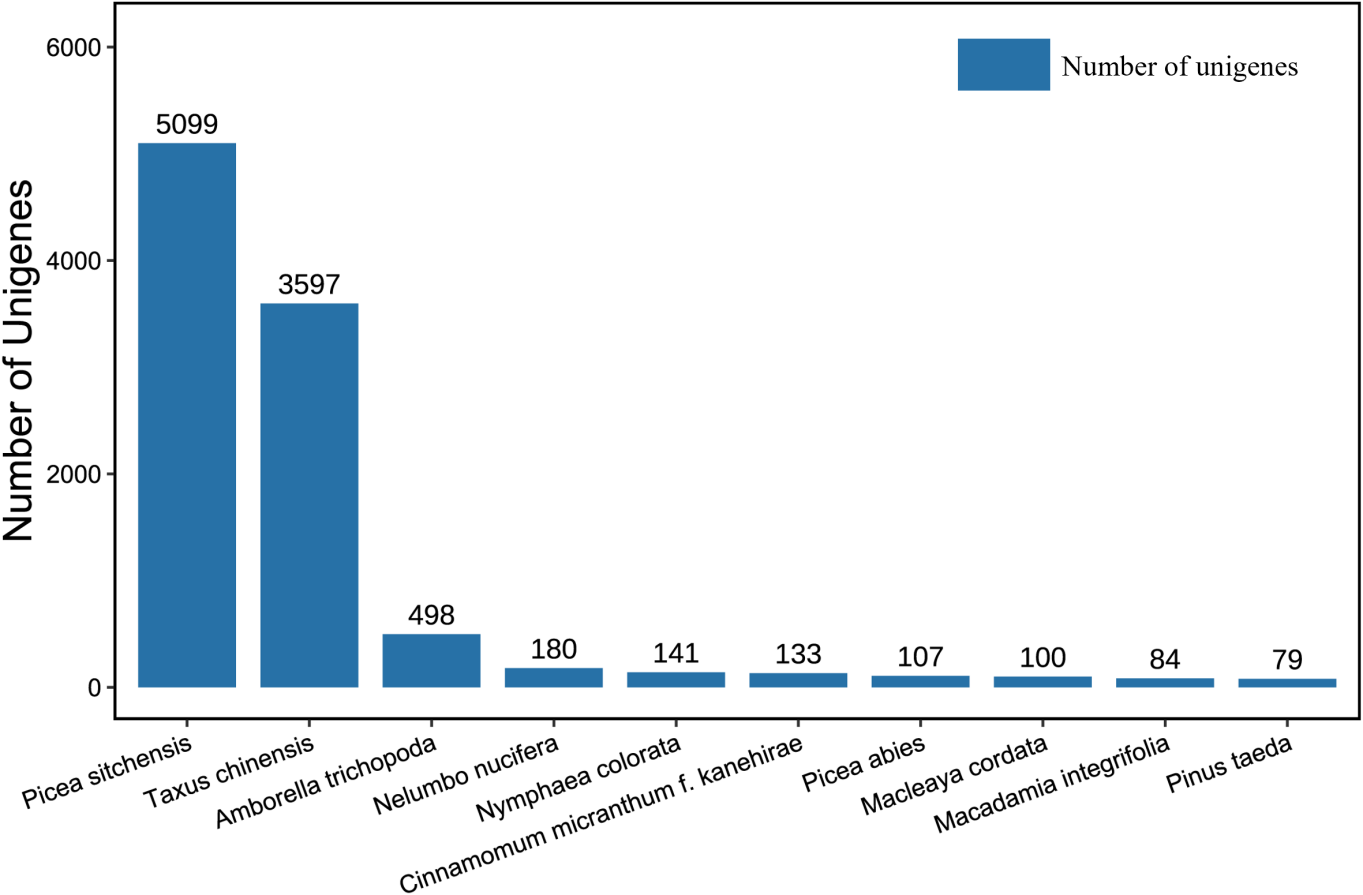
The top ten Nr homologous species of transcripts compared to *Picea mongolica*. Nr, Non-redundant protein.

The 12,092 transcripts annotated from the Nr database were compared with 253 species (**Supplementary Table 1**). **Figure 4** displays the top 10 species by number of homologous sequences. Among them, the number of genes compared to *Picea sitchensis* and *Taxus chinensis* was the highest, at 5,099 and 3,597, respectively. The next in line were plants such as *Amborella trichopoda*, *Nelumbo nucifera*, and *Nymphaea colorata*, etc. Among the transcripts, the species most similar to *P. mongolica* was *P. sitchensis*.

*P. sitchensis* is distributed along the Pacific coast of North America, whereas *P. mongolica* is endemic to the Hunshandake Sandy Land in Inner Mongolia, China. Despite their vast geographical separation, molecular phylogenetic studies have revealed that spruce species from western North America and those from East Asia form a closely related clade. These species share a relatively recent common ancestor, which accounts for the high degree of sequence similarity observed in their genomes. In contrast, *P. abies*, the predominant spruce species in Europe, belongs to a distinct evolutionary lineage. It diverged earlier from the East Asian-North American clade, and prolonged independent evolution has resulted in greater accumulation of genomic divergence, leading to reduced sequence similarity in orthologous genes. Consequently, only 107 highly similar transcripts were identified. Although *P. abies* is currently the only species in the genus Picea with a published reference genome (Nystedt et al., 2013), the initial genome assembly suffers from relatively low completeness and annotation quality, thereby limiting its utility as a reference for phylogenetically distant species such as *P. mongolica*.

### 3.3 GO and KOG annotation

**Figure 5:**
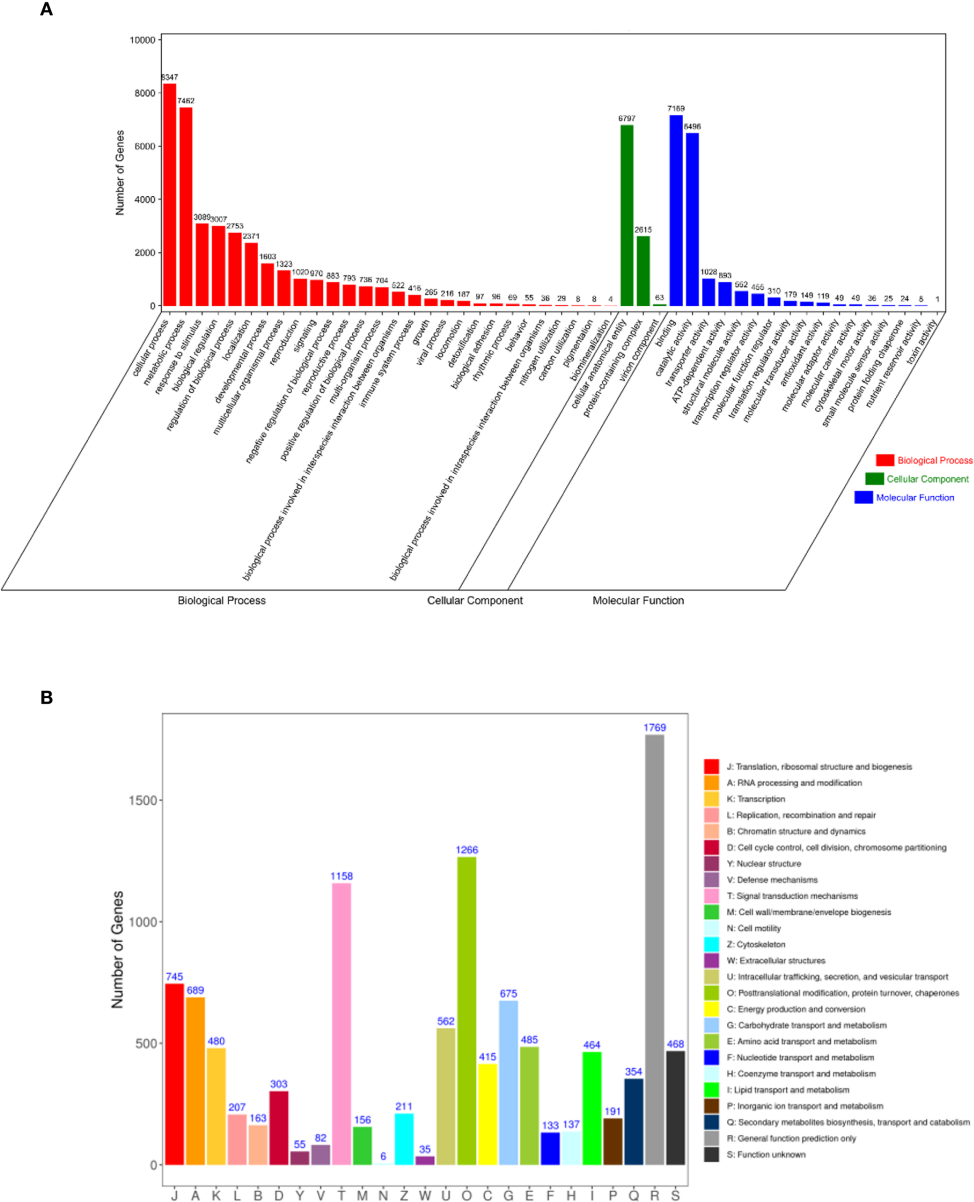
GO and KOG enrichment analyses of *Picea mongolica* transcripts. **(A)** GO term classifications of transcript sequences. **(B)** KOG annotation of transcript sequences. GO, Gene Ontology; KOG, Eukaryotic Orthologous Groups.

Functional annotation analysis successfully assigned 10,564 transcripts to GO categories that were classified into 48 functional groups across three major domains: biological processes, cellular components, and molecular functions (**Figure 5A**). Within the biological process domain, cellular process (8,347), metabolic process (7,462), and responses to stimulus (3,089) were the most abundant categories. The cellular component domain was dominated by cellular anatomical entity (6,797), followed by protein-containing complex (2,615) and virion components (63). In the molecular function classification, binding (7,169) was the most prevalent category, with catalytic activity (6,496) and transporter activity (1,028) being the subsequent major groups. A total of 8,444 transcripts were functionally annotated in the KOG database and classified into 25 distinct categories (**Figure 5B**). The most abundant functional category was general function prediction (1,769), followed by post-translational modifications, protein turnover, chaperones (1,266), and signal transduction mechanisms (1,158). In contrast, cell motility was the least populated category, with only six annotated transcripts. The predominance of transcripts related to response to stimulus and signal transduction mechanisms is particularly noteworthy, as it may reflect the inherent stress adaptation mechanisms of *P. mongolica*, a species native to a harsh desert environment, which could be co-opted during the *in vitro* stress of SE.

### 3.4 Analysis of KEGG pathway annotation

The KEGG annotation analysis successfully assigned 11895 P*. mongolica* transcripts to 134 metabolic pathways across five major categories (**Supplementary Table 2**). The metabolism category had the highest number of annotated genes (8,832), followed by genetic information processing (1,583), cellular processes (319), environmental information processing (299), and organism systems (213). Among the specific metabolic pathways, metabolic pathways (2,187) represented the most abundant category, with biosynthesis of secondary metabolites (1,301), carbon metabolism (431), and biosynthesis of amino acids (353) also being prominently represented within the metabolism category (**Table 1**). Notably, previous studies have demonstrated that carbon source type and concentration significantly influence SE, playing a crucial role among the three key regulatory factors (plant hormones, carbon sources, and nitrogen sources) in SE. The significant enrichment of pathways such as carbon metabolism and starch and sucrose metabolism underscores the massive metabolic reprogramming and high energy demand required for the rapid cell proliferation and differentiation that characterizes SE.

**Table 1:** Kyoto Encyclopedia of Genes and Genomes pathway enrichment.

Pathway	The number and proportion of genes	Pathway ID
Metabolic pathways	2187 (53.45%)	ko01100
Biosynthesis of secondary metabolites	1301 (31.79%)	ko01110
Carbon metabolism	431 (10.53%)	ko01200
Biosynthesis of amino acids	353 (8.63%)	ko01230
Protein processing in endoplasmic reticulum	245 (5.99%)	ko04141
Glycolysis / Gluconeogenesis	229 (5.6%)	ko00010
Starch and sucrose metabolism	193 (4.72%)	ko00500
Pyruvate metabolism	186 (4.55%)	ko00620
Spliceosome	186 (4.55%)	ko03040
Ribosome	171 (4.18%)	ko03010
Amino sugar and nucleotide sugar metabolism	166 (4.06%)	ko00520
mRNA surveillance pathway	161 (3.93%)	ko03015
Plant-pathogen interaction	158 (3.86%)	ko04626
Plant hormone signal transduction	157 (3.84%)	ko04075
Endocytosis	150 (3.67%)	ko04144
Cysteine and methionine metabolism	142 (3.47%)	ko00270
Glyoxylate and dicarboxylate metabolism	140 (3.42%)	ko00630
RNA degradation	130 (3.18%)	ko03018
Citrate cycle (TCA cycle)	123 (3.01%)	ko00020
Nucleocytoplasmic transport	122 (2.98%)	ko03013

### 3.5 Analysis of the long-read transcript structure

**Figure 6:**
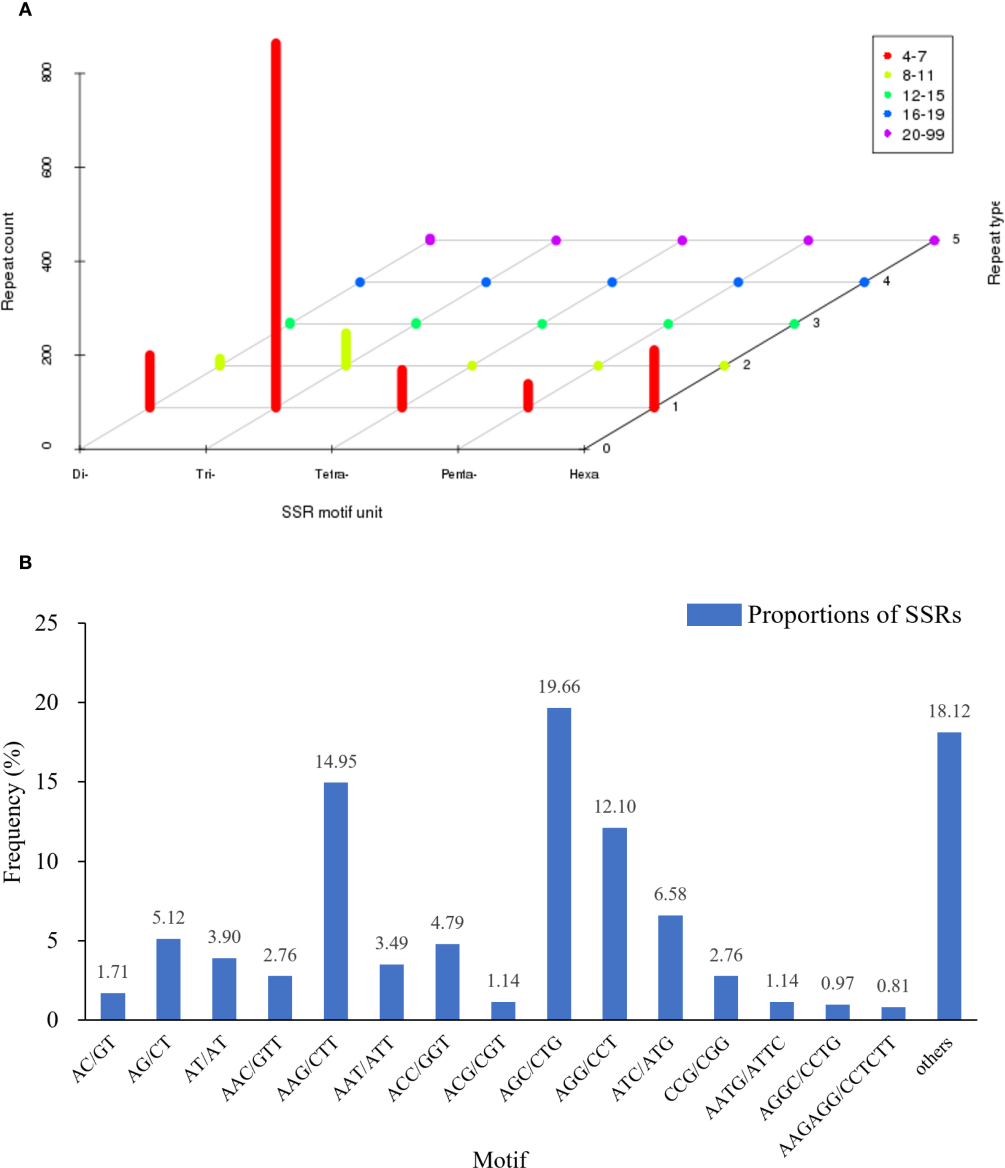
Distribution of SSR nucleotide classification in *Picea mongolica* transcriptome. **(A)** Distribution statistics of six types of SSRs. **(B)** Proportions of SSRs with different types among tandem repeat elements in total SSRs.

To develop molecular markers for future genetic studies and breeding applications in this endangered species, we identified and characterized SSR loci within the transcriptome assembly. SSR analysis of the 12,232 isoforms from *P. mongolica* using MISA1.0 identified 1,006 sequences (8.22%) containing SSR loci. Among these, 159 sequences harbored two or more SSR loci, with 1,231 SSR loci detected, including di-, tri-, tetra-, penta-, and hexanucleotide repeats (**Figure 6A**). Trinucleotide repeats were the most abundant (846), with AGC/CTG (242, 19.66%), AAG/CTT (184, 14.95%), and AGG/CCT (149, 12.10%) being the predominant motifs (**Figure 6B**). Additionally, 123 sequences contained compound SSR loci. The majority of repeats (1,138) exhibited 4-7 repeat units, followed by 8-11 repeats (84).

**Figure 7:**
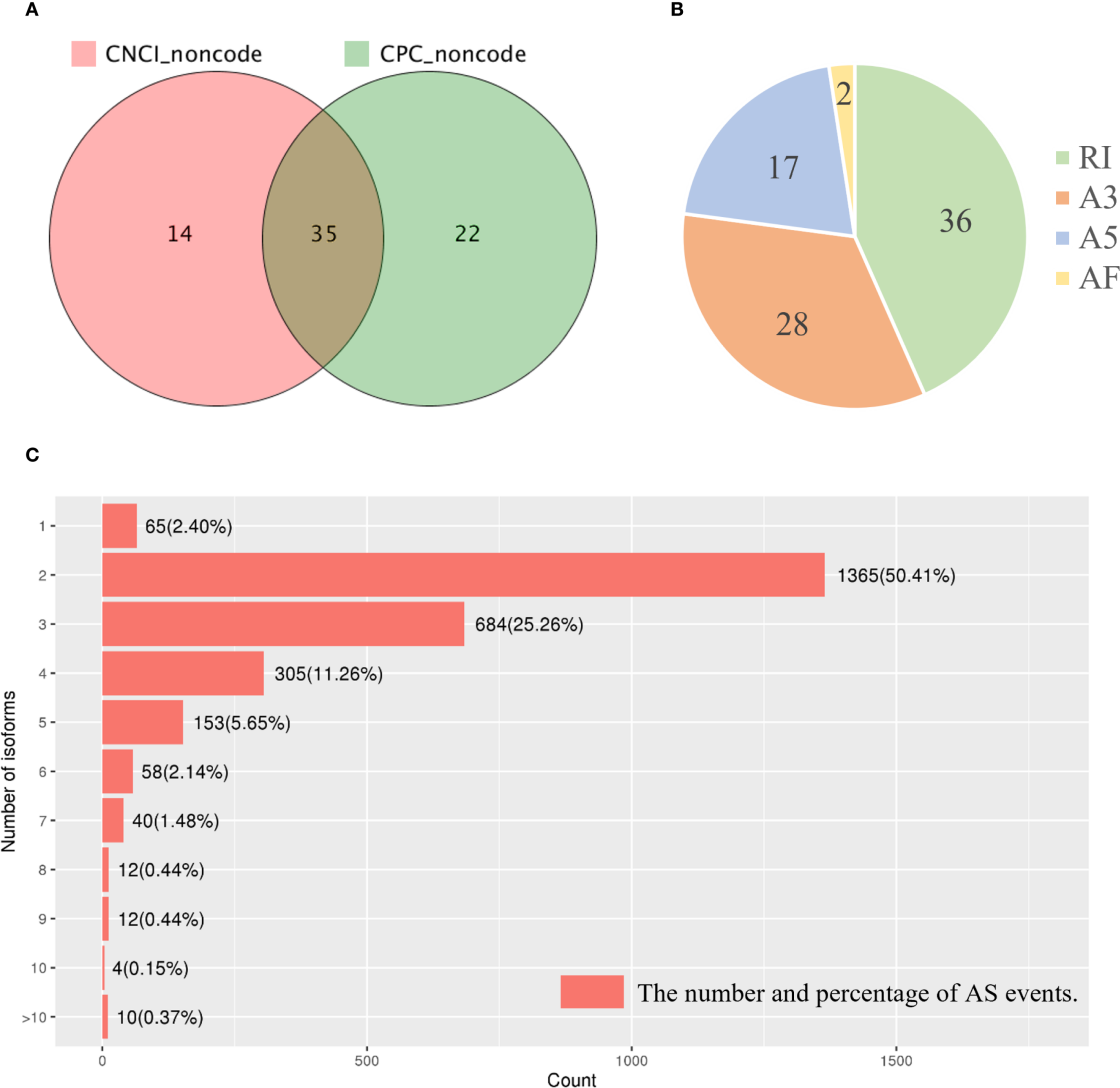
Analysis of the candidate lncRNAs and AS events in the *Picea mongolica* transcriptome. **(A)** Candidate lncRNAs identified by CNCI and CPC. **(B)** Statistics of the isoforms number. A3, alternative 3’splice site; A5, alternative 5’splice site; AF, alternative first exon; RI, retained intron; SE, skipping exon; AS, alternative splicing. **(C)** The number and percentage of AS events.

As demonstrated in Section 3.2, most of the long-read sequences were well annotated. For the remaining unannotated transcripts, a coding potential assessment using both CNCI and CPC software identified 35 putative lncRNAs (**Figure 7A**). We used INFERNAL for multiple sequence alignment, secondary structure prediction, and covariance modeling based on conserved sequences and structural features. However, none of the 35 lncRNAs exhibited significant matches. This lack of annotation may be explained by the high sequence divergence of lncRNAs, particularly in conifers such as *P. mongolica*, which are evolutionarily distant from the model plants well-represented in current databases. It is likely that many of these lncRNAs are novel and species-specific, possibly involved in lineage-specific regulatory processes during SE. Furthermore, existing databases remain biased toward model organisms, limiting homology-based detection in non-model species. These results suggest that *P. mongolica* may possess a set of previously uncharacterized, rapidly evolving lncRNAs that could play roles in its unique embryogenic programming. Future functional studies are needed to clarify their biological significance.

AS analysis with SUPPA software revealed 83 AS events belonging to four distinct types: two alternative first exons, 17 alternative 5’ splice sites, 28 alternative 3’ splice sites, and 36 intron retentions (**Figure 7B**). Furthermore, the research revealed that 65 (2.40%) unigenes had only one isoform. 1,365 (50.41%), 684 (25.26%), and 305 (11.26%) genes included two, three, and four isoforms, respectively. Ten (0.37%) genes were discovered in more than ten splice isoforms (**Figure 7C**). To further explore the biological relevance of these AS events, we performed functional enrichment analysis on the affected transcripts. KEGG pathway analysis revealed that these isoforms were significantly enriched in Starch and sucrose metabolism and Sphingolipid metabolism (**Supplementary Figure 1**). The enrichment of starch and sucrose metabolism is particularly noteworthy, as it aligns with our previous transcriptomic and metabolomic findings (Dai et al., 2025) and underscores the critical role of carbon source reprogramming in supporting the high energy demands of SE. Additionally, GO analysis showed enrichment in terms related to photosynthesis (e.g., photosystem I/II) and cellular components like the thylakoid membrane, suggesting a role for AS in the metabolic restructuring that accompanies embryogenic development.

### 3.6 Prediction of transcription factor families

**Figure 8:**
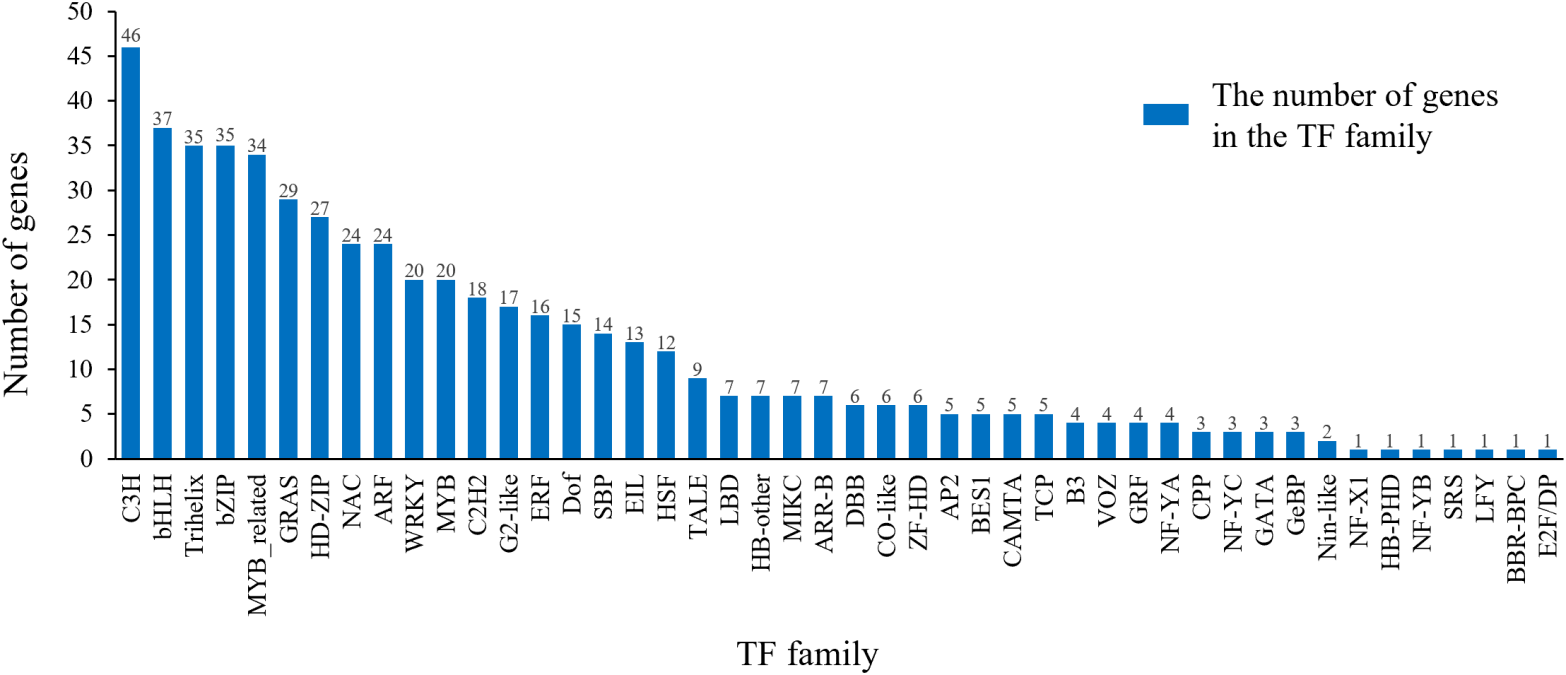
Transcription factor family analysis.

Through a comprehensive analysis of the long-read transcriptome sequencing data, we identified 548 TFs belonging to 46 distinct TF families from 12,232 long-read transcripts using hmmScan alignment against the TF database (**Supplementary Table 3**). The C3H family was the most abundant TF group (46, 8.39%), followed by bHLH (37, 6.75%), trihelix (35, 6.39%), bZIP (35, 6.39%), and MYB-related (34, 6.20%) families (**Figure 8**). Beyond the overall catalog of TFs, we sought to identify key regulators specifically associated with the acquisition of embryogenic competence. By analyzing expression levels across stages, we found that in addition to AP2, several TFs from the NAC, NF-Y, and LBD families exhibited pronounced upregulation specifically in the EC compared to NEC (**Supplementary Table 4**). For instance, ABI4 expression increased more than 104 fold in EC, suggesting their potential as novel candidates involved in initiating SE.

### 3.7 Cloning and bioinformatics analysis of the *PmBBM*

Based on the long-read transcriptome sequencing data, a 2,367 bp gene encoding 788 amino acids was identified through screening and comparative analysis (GenBank accession number PV941855) (**Supplementary Figure 2**), and designated as *PmBBM*. The encoded protein of *PmBBM* has a molecular weight of 86,633.16 Da and an isoelectric point of 5.85. There were 82 negatively charged residues and 68 positively charged residues. The amino acid with the highest content is serine, which accounts for 12%, whereas cysteine and tryptophan were the least abundant, accounting for 1% (**Supplementary Figure 3**). The molecular formula of the protein was determined to be C_3699_H_5766_N_1104_O_1240_S_34_, with a total of 11,843 atoms. The aliphatic index of the protein was 61.50, indicating high stability.

The results of the hydrophilicity/hydrophobicity indicated that most regions scored negative values, suggesting predominant hydrophilicity (**Supplementary Figure 4A**). Transmembrane domain prediction revealed the absence of transmembrane helices in PmBBM (**Supplementary Figure 4B**). Signal peptide analysis confirmed the absence of signal peptides in this protein (**Supplementary Figure 4C**).

**Figure 9:**
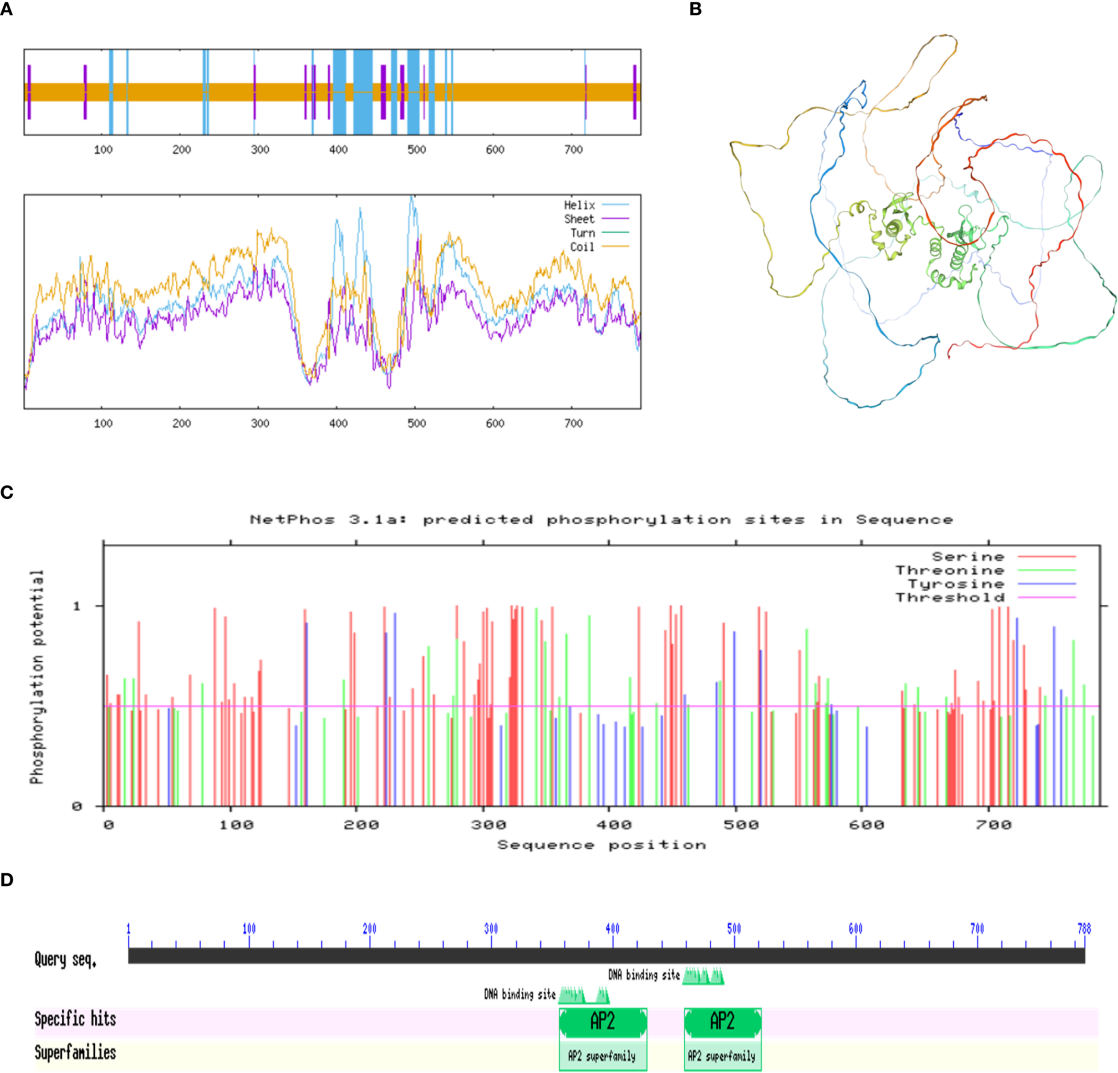
Analysis of the PmBBM protein. **(A)** Secondary structure of PmBBM. **(B)** Tertiary structure of PmBBM. **(C)** Prediction of phosphorylation sites in PmBBM. **(D)** Conserved domain analysis of PmBBM. Scale.

The predicted secondary structure of the PmBBM protein (**Figure 9A**) consisted of 13.96% α-helices, 5.71% extended strands, and 80.33% random coils. For tertiary structure prediction (**Figure 9B**), a 3D model of PmBBM was constructed using a template (PDB ID: A0A088BUC1.1. A), exhibiting a Global Model Quality Estimation (GMQE) score of 0.45 and 93.27% sequence similarity to AP2 domain-containing proteins. These results indicated a structurally diverse protein with abundant coils and helices. Phosphorylation site prediction (**Figure 9C**) revealed 107 potential sites, including 27 threonine, 71 serine, and 9 tyrosine residues. Furthermore, a conserved domain analysis using SMART (**Figure 9D**) demonstrated that PmBBM contained two AP2 domains.

**Figure 10:**
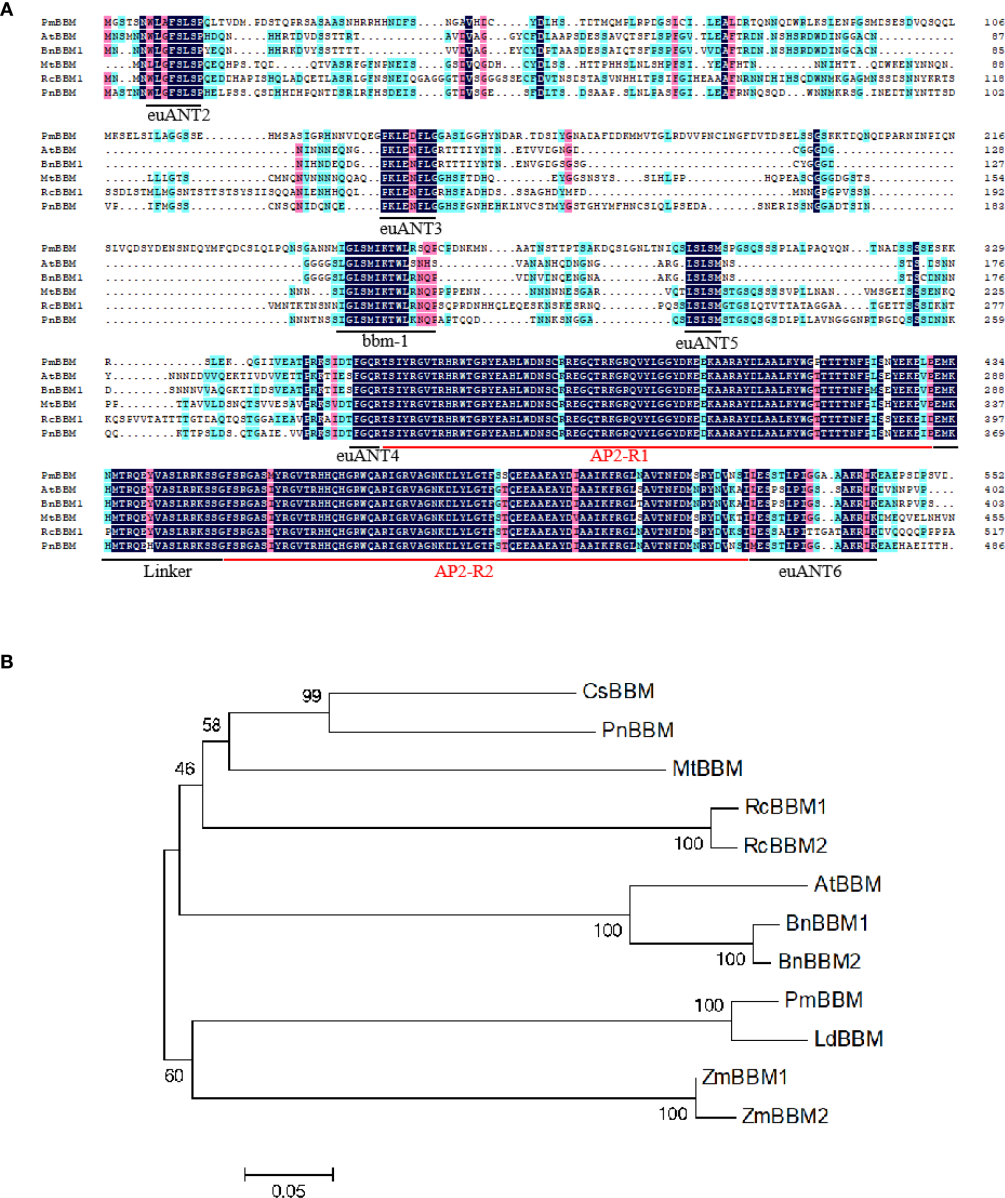
The comparison of the deduced amino acid sequences and the phylogenetic tree analysis of PmBBM. **(A)** The comparison of the deduced amino acid sequences of PmBBM and other BBM proteins. The numbers indicate the amino acid positions in the context of the entire protein. The euANT2, euANT3, euANT4, euANT5, euANT6, AP2-R1, Linker, AP2-R2, and bbm-1 are underlined. **(B)** The phylogenetic tree analysis of PmBBM and other BBM proteins. The tree was constructed by the neighbor-joining method with the MEGA program. Branch numbers represent the percentage of bootstrap values in 1000 sampling replicates and the scale indicates branch lengths.

The deduced amino acid sequences of PmBBM was compared to other BBM proteins from *A. thaliana*, *B. napus*, *Medicago truncatula*, *R. canina*, and *Populus nigra* using the DNAMAN software. PmBBM possesses two AP2 domains (AP2-R1 and AP2-R2) and a linker region lying between them (linker), indicating that PmBBMs belong to the AP2 family. Moreover, RcBBMs contained the bbm-1 motif, and all motifs were conserved in the euANT lineage: euANT2, euANT3, euANT4, euANT5, and euANT6 (**Figure 10A**). The phylogenetic tree revealed that PmBBM has the closest genetic relationship with *Larix decidua* BBM (**Figure 10B**). This close clustering with a BBM ortholog from another conifer species strongly supports the identity of our cloned gene and suggests evolutionary conservation of its function in conifer embryogenesis.

### 3.8 Subcellular localization of *GFP-PmBBM* in transiently transformed tobacco

**Figure 11:**
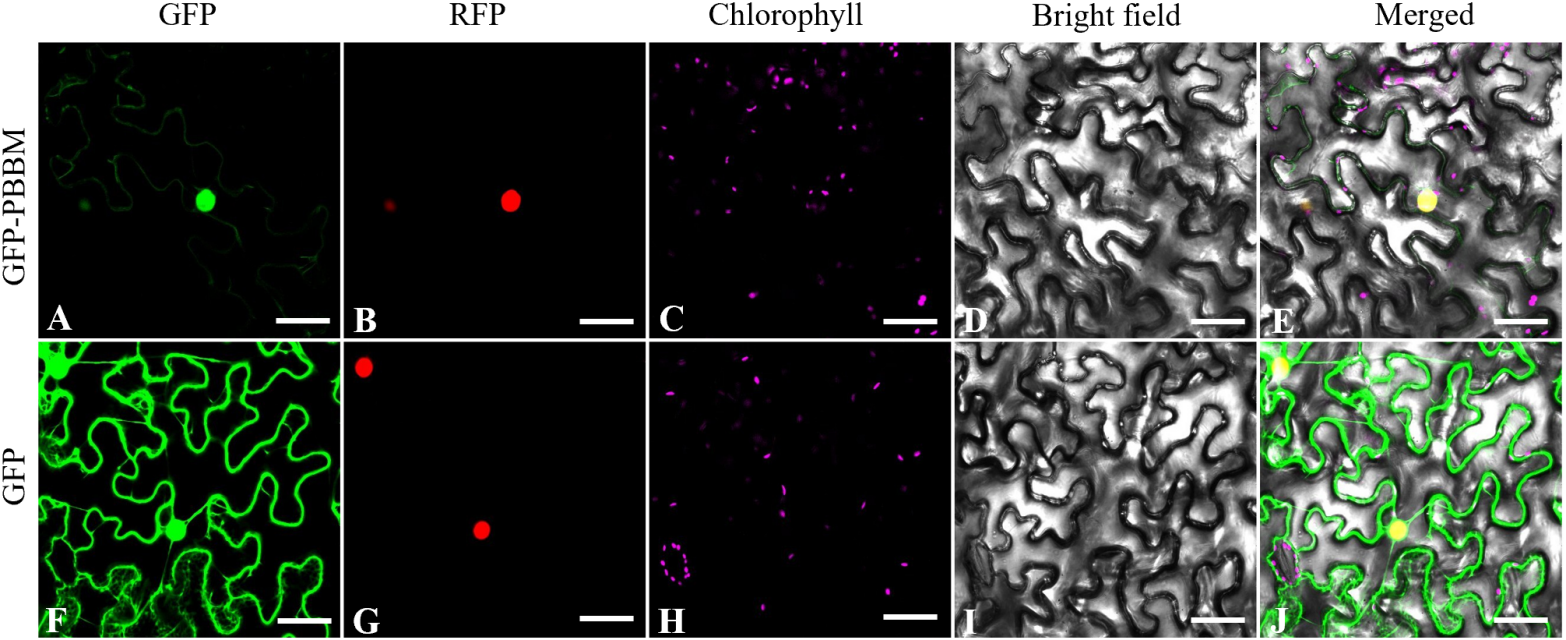
The subcellular localization of PmBBM in tobacco. Fluorescence images **(A, F)**, nuclear marker **(B, G)**, chlorophyll **(C, H)**, bright-field images **(D, I)**, and the merged images **(E, J)**. bars = 100 μm.

To determine the precise subcellular localization of PmBBM, we developed the fusion expression vector p35S::PmBBM-GFP, which enables the expression of the fusion protein in tobacco leaves through *Agrobacterium tumefaciens*-mediated delivery. The findings depicted in **Figure 11** shows that the fluorescence signal of p35S::PmBBM corresponded to that of the cell nucleus marker RFP. As a result, it is expected to be largely found in the nucleus, with minimal expression in the cytoplasm.

### 3.9 *PmBBM* and AP2 transcription factor family expression profiling during SE in *P. mongolica*

**Figure 12:**
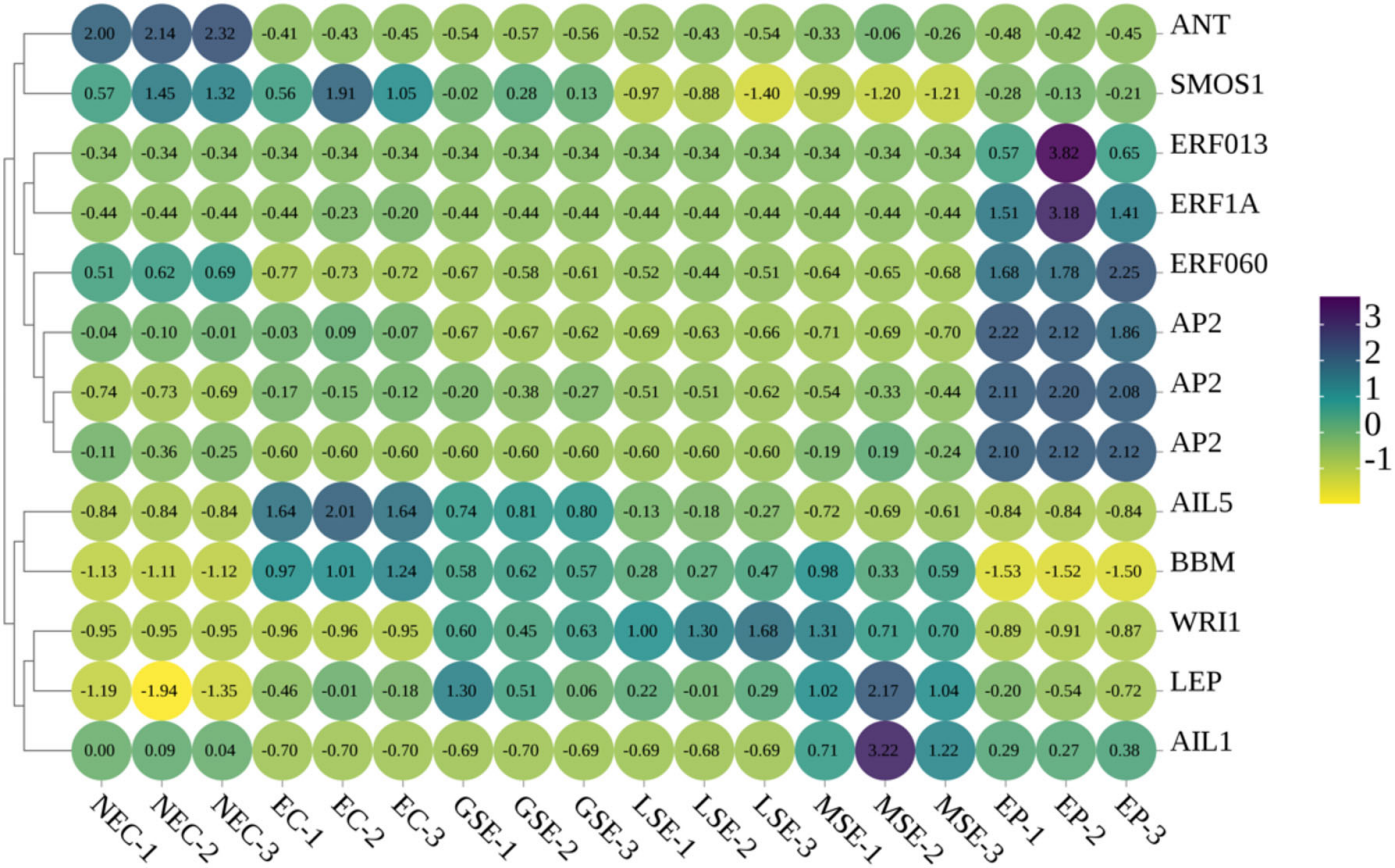
Differences in the expression levels of the *PmBBM* and AP2 families of genes in NEC, EC, GSE, LSE, MSE, and EP. The color scale represents Z-score of FPKM. The number in each grid represents the Z-score value. NEC, non-embryogenic callus; EC, embryogenic callus; GSE, global somatic embryos; LSE, late somatic embryos; MSE, mature somatic embryos; EP, somatic embryo-derived plantlets.

Based on our transcriptome sequencing results for *P. mongolica* SE at key stages, we analyzed the expression patterns of *PmBBM* and other AP2 transcription factor family genes in NEC, EC, GSE, LSE, MSE, and EP (**Figure 12**). *PmBBM* was shown to be strongly expressed during somatic embryo formation and development, with the highest levels in EC; however, its expression was minimal in NEC and EP. The AP2 family of genes displays two distinct expression patterns: genes highly expressed during SE, such as *BBM*, *WRI1*, and *AIL5*; and genes predominantly expressed in NEC or EP, including *ANT*, *EPF*, and *AP2*. Our expression analysis reveals that specific AP2/ERF genes (particularly *PmBBM*) are dynamically expressed during SE in *P. mongolica*, suggesting their potential roles in regulating this specific developmental process.

## 4 Discussion

This study was designed to elucidate the molecular underpinnings of SE in the endangered conifer *P. mongolica*, with a particular focus on identifying key regulatory genes. By employing PacBio SMRT sequencing, we established a foundational transcriptome resource that enabled us to successfully clone and characterize the dynamic expression of *PmBBM*, a central transcription factor in SE. Phylogenetic analysis revealed that PmBBM shares the closest evolutionary relationship with the BBM protein from *Larix decidua*, another conifer species. This high degree of conservation suggests that the embryogenic function of BBM is likely shared among conifers, providing a strong phylogenetic basis for inferring *PmBBM*’s role in SE. Furthermore, expression profiling demonstrated that *PmBBM* is predominantly expressed during early somatic embryo formation, consistent with its putative role as an inducer of embryogenic development.

These findings complements our previous work on stage-specific expression dynamics during SE by providing the essential genomic infrastructure for future functional studies. The PacBio long-read sequencing approach employed here overcomes the inherent limitations of short-read assemblies in distinguishing between closely related paralogs and alternatively spliced isoforms. We have accurately identified: 12,232 high-quality long-read transcript sequences, which serve as a definitive reference for gene annotation; 83 alternative splicing events and 35 lncRNAs, which were previously uncharacterized; and the complete coding sequence (CDS) and structure of key regulators like PmBBM, enabling its functional cloning and characterization. The stage-specific expression patterns of PmBBM, analyzed using the reference generated here, are consistent with and extend our previous findings, highlighting the sustained importance of this TF family throughout SE. The SSR markers and lncRNAs identified herein provide a new set of tools and targets for further investigating the mechanisms uncovered in our prior multi-omics study.

In a comparable study, Li et al. (2020) employed an integrated NGS-TGS approach to analyze pooled samples (23 specimens across 10 developmental stages) from *Bletilla striata* suspension cultures. Their study generated 100,276 high-quality long-read transcripts, including 53,316 KOG-annotated unigenes (classified into 26 functional categories) and 8,020 KEGG-mapped unigenes (assigned to 363 pathways). Additionally, they identified 15,133 lncRNAs and 68,996 SSR-containing coding sequences. Similar long-read transcriptome investigations have been reported for diverse plant species, including *Platycladus orientalis* (Liao et al., 2024), *Solanum tuberosum* (Yan et al., 2022), and *Lilium pumilum* (Song et al., 2020). As demonstrated in these studies, TGS has emerged as a powerful tool for developing genomic resources and molecular markers in non-model plants.

AS is a key mechanism that regulates gene expression and protein variety, as well as improving transcriptome complexity and modifying developmental processes (Szakonyi and Duque, 2018). During SE, AS participates in critical biological processes including cell fate determination, morphogenesis, and signal transduction. Du et al. (2019) systematically analyzed AS patterns across different stages of callus induction in maize, identifying over 2,000 splicing events per stage. Notably, the genes involved in spliceosome assembly, metabolic pathways, and mRNA surveillance exhibited pronounced AS dynamics during callus induction. Their findings revealed that AS cooperates with transcriptional regulation to facilitate callus formation. Similarly, in *Larix kaempferi*, microRNA171 and its target gene *LaSCL6* generate two alternatively spliced isoforms that regulate SE (Zang et al., 2024). Studies on *Panax ginseng* have further demonstrated that the *PgCDPK2d* subfamily, particularly its alternatively spliced variants, functionally contributes to the SE (Kiselev et al., 2009). In this study, PacBio long-read transcriptome sequencing identified 188 isoforms exhibiting AS during SE in *P. mongolica*. KEGG analysis found that these genes were primarily enriched in pathways such as photosynthesis, starch and sucrose metabolism, and sphingolipid metabolism (**Supplementary Figure 1**). In the transcriptome and metabolome studies during the SE in *P. mongolica*., we also found that the differentially expressed genes and metabolites were significantly enriched in the starch and sucrose synthesis pathways (Dai et al., 2025).

To address the stage-specificity of metabolic pathways, we examined the expression patterns of genes within the plant hormone signal transduction pathway. Although the PacBio sequencing was performed on a pooled sample, our stage-specific RNA-seq data (**Supplementary Figure 5**) revealed distinct phytohormone dynamics: auxin and cell growth response genes (e.g., *XTH, LAX*) were predominantly expressed in the EC and NEC stages, consistent with their roles in promoting cell division and embryogenic induction. In contrast, genes associated with abscisic acid (ABA) and gibberellic acid (GA) signaling (e.g., GAI, PYL, SRK) showed elevated expression in the GSE and MSE stages, aligning with their established functions in somatic embryo maturation and preparation for desiccation tolerance.

The broader functional enrichment analysis of the entire transcriptome further illuminated the key biological processes underpinning SE in this desert-adapted conifer. The significant enrichment of pathways such as carbon metabolism, starch and sucrose metabolism, and biosynthesis of amino acids (**Table 1**) highlights a massive demand for energy and biosynthetic precursors to drive the rapid cell proliferation and differentiation characteristic of embryogenic development. This is particularly relevant for *P. mongolica*, a species endemic to the nutrient-poor Hunshandake Sandy Land, suggesting an efficient carbon allocation mechanism is crucial for SE success. Furthermore, the enrichment of plant hormone signal transduction pathways underscores the well-established central role of phytohormones like auxins and cytokinins in initiating and sustaining SE. Notably, the detection of enriched stress-responsive GO terms (e.g., response to stimulus, **Figure 5A**) may be intrinsically linked to the species’ evolution in a harsh desert environment. The inherent stress tolerance mechanisms of *P. mongolica* might be co-opted during the *in vitro* SE process, which itself imposes significant osmotic and oxidative stresses on tissues.

Key transcription factors serve as primary mediators of SE by initiating and regulating downstream gene expression in response to phytohormone signals such as auxins and cytokinins, thereby triggering embryogenic transitions and controlling somatic embryo development (Asghar et al., 2023). Several AP2/ERF family members regulate somatic embryogenesis. The Arabidopsis *ERF* homolog *MtSERF1*, an ethylene-inducible gene in *Medicago sativa*, is expressed in rapidly proliferating embryogenic tissues, somatic embryos, and zygotic embryos. *MtSERF1* knockout significantly suppressed somatic embryo regeneration (Mantiri et al., 2008). Another AP2/ERF member, *EMK* is specifically expressed in Arabidopsis zygotic embryos and maintains embryogenic cell identity. Ectopic expression of *EMK* in Arabidopsis cotyledons induces SE (Tsuwamoto et al., 2010).

Among AP2/ERF members, the *BBM* gene has been the most extensively studied (Boutilier et al., 2002). The embryogenic properties of BBM have been successfully used to improve the *in vitro* regeneration and transformation systems in crops (Deng et al., 2009; Florez et al., 2015). Co-overexpression of maize *BBM* and *WUS* genes in immature embryo transformation systems significantly increases the success rate of transgenic calli, with most producing healthy, fertile plants (Lowe et al., 2016). The ectopic expression of rice *BBM1* in unfertilized egg cells can induce parthenogenesis (Khanday et al., 2019). Recent studies have identified *LEC/AGL15* genes as positive regulators in SE, with BBM functioning as a transcription factor that directly activates *LEC1*, *LEC2*, and *FUS3* transcription (Salaün et al., 2021). Our successful cloning and characterization of *PmBBM* establishes a crucial foundation for future functional studies on this gene during SE in this conifer species.

Although this work established the reference-quality transcriptome resource for *P. mongolica* and provides initial insights into the expression dynamics of key regulators (e.g., *PmBBM*) during SE, several limitations must be considered. First, the absence of a reference genome restricted our analysis to the transcriptome level, potentially overlooking crucial non-coding regulatory elements and unexpressed genes. Additionally, the sampling strategy focused solely on specific stages of SE, thereby limiting a comprehensive understanding of developmental processes and stress responses under natural conditions. Future investigations should prioritize the assembly of chromosome-level reference genomes and employ multi-omics approaches (including metabolomics, proteomics, and epigenomics) to systematically decipher the molecular mechanisms underlying desert adaptation in *P. mongolica*. Complementary functional validation of key genes through CRISPR-based editing or overexpression systems, coupled with the development of molecular markers for assisted breeding, will substantially contribute to the conservation and sustainable use of this endangered conifer species. The identification of 1,006 SSR loci within the transcriptome offers a valuable set of molecular markers for future population genetics studies, assessing genetic diversity, and supporting marker-assisted breeding programs in *P. mongolica*.

## 5 Conclusion

This study successfully obtained sequence and structural information on the long-read transcripts during SE in *P. mongolica* using SMRT sequencing. The transcript sequences were subjected to comprehensive analysis, which included KOG, GO, and KEGG enrichment studies. Moreover, AS events, TFs, lncRNAs, and SSRs were systematically predicted. Based on these findings, the *PmBBM* gene was cloned and bioinformatically characterized. Furthermore, expression patterns during SE were analyzed. These findings not only enrich the genetic database of *P. mongolica* but also provide a scientific foundation for identifying key regulatory genes, molecular biological investigations, and modern breeding programs related to SE in this species.

## Data Availability

The datasets presented in this study can be found in online repositories. The names of the repository/repositories and accession number(s) can be found in the article/**Supplementary Material**.

## Supplementary material

Supplementary Figure 1 Functional enrichment analysis of alternatively spliced transcripts during somatic embryogenesis in *P. mongolica*.

Supplementary Figure 2 CDS sequence of *PmBBM* gene and its encoded amino acid sequence.

Supplementary Figure 3 The amino acid compositon and content of PmBBM protein.

Supplementary Figure 4 Analysis of PmBBM protein. **(A)** Analysis of hydrophobicity/ hydrophilicity of the PmBBM protein. **(B)** Prediction of transmembtane structure of the protein. **(C)** Analysis of signal peptide of the PmBBM protein.

Supplementary Figure 5 Expression dynamics of plant hormone signal transduction genes during somatic embryogenesis.

Supplementary Table 1 Statistical analysis of isoform distribution among diverse species.

Supplementary Table 2 Functional annotation of isoforms by KEGG database alignment.

Supplementary Table 3 Statistical analysis of transcription factors and their families.

Supplementary Table 4 Top 20 transcription factors with the highest expression in embryogenic callus.
