# Supplementary figures and images for: PacBio single-molecule long-read transcriptome sequencing and analysis of somatic embryogenesis in Picea mongolica
Source: Front Plant Sci. 2025 Oct 8;16:1682365. doi: 10.3389/fpls.2025.1682365 (PMC12540384; doi:10.3389/fpls.2025.1682365)

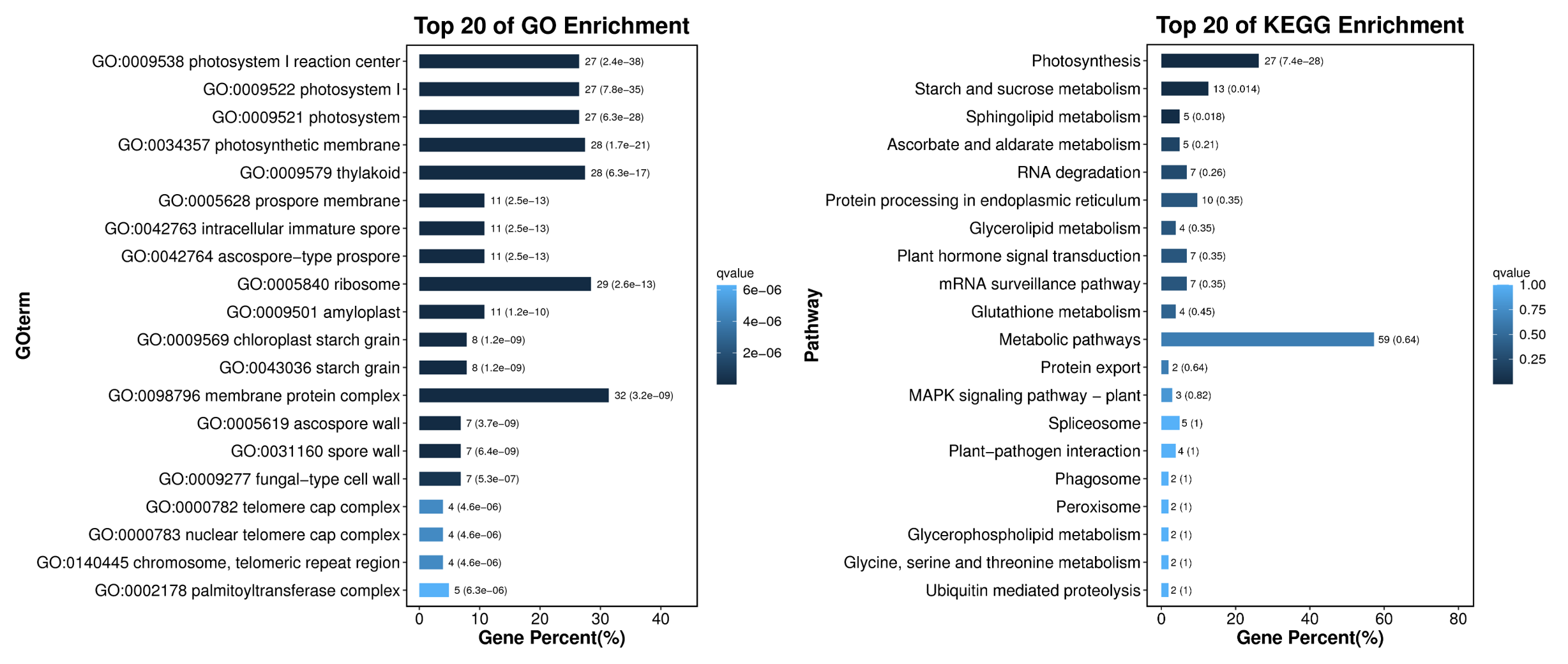

Supplement: Supplementary Figure 1 — Functional enrichment analysis of alternatively spliced transcripts during somatic embryogenesis in P. mongolica. [file Image1.tif]

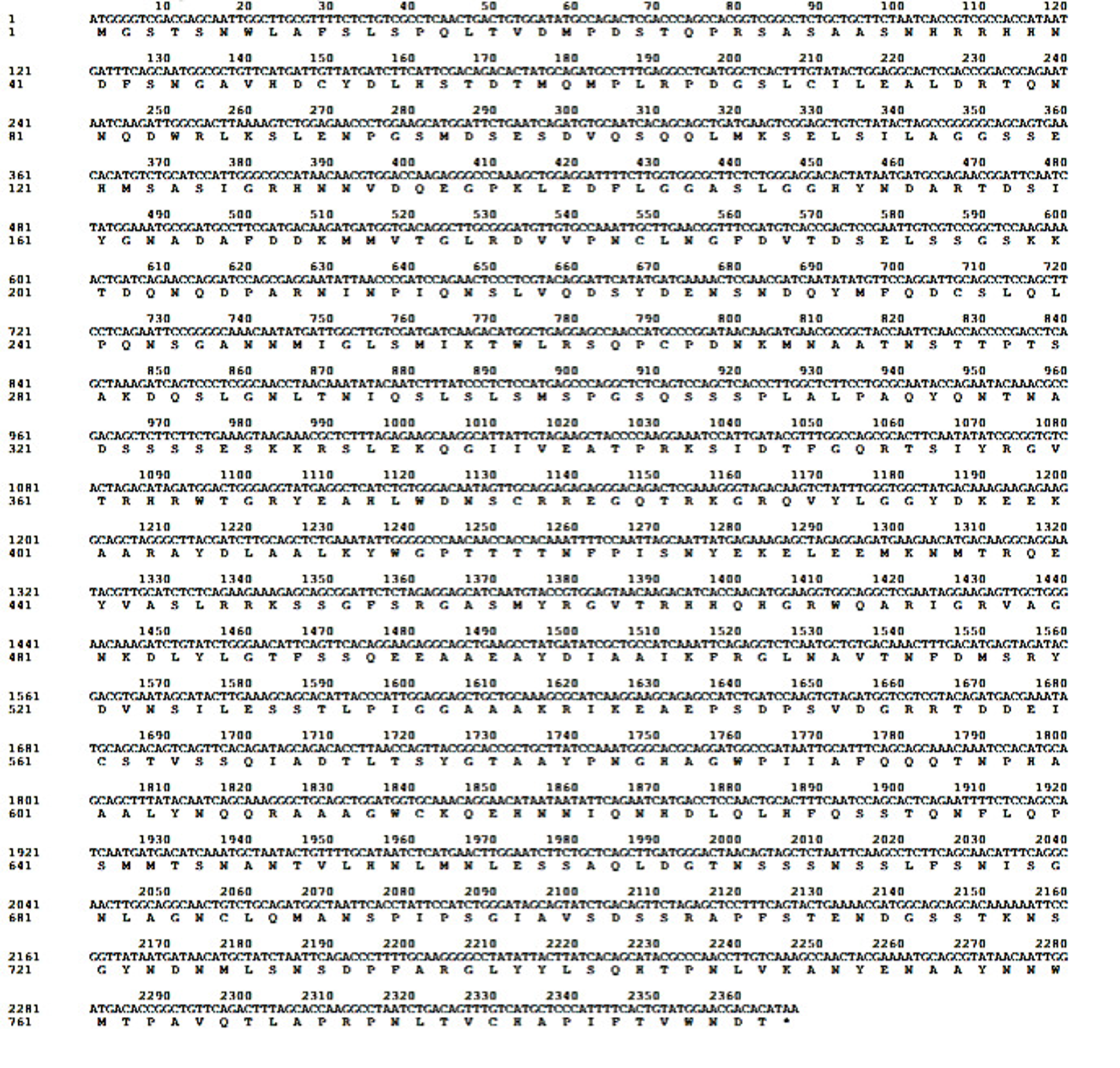

Supplement: Supplementary Figure 2 — CDS sequence of PmBBM gene and its encoded amino acid sequence. [file Image2.tif]

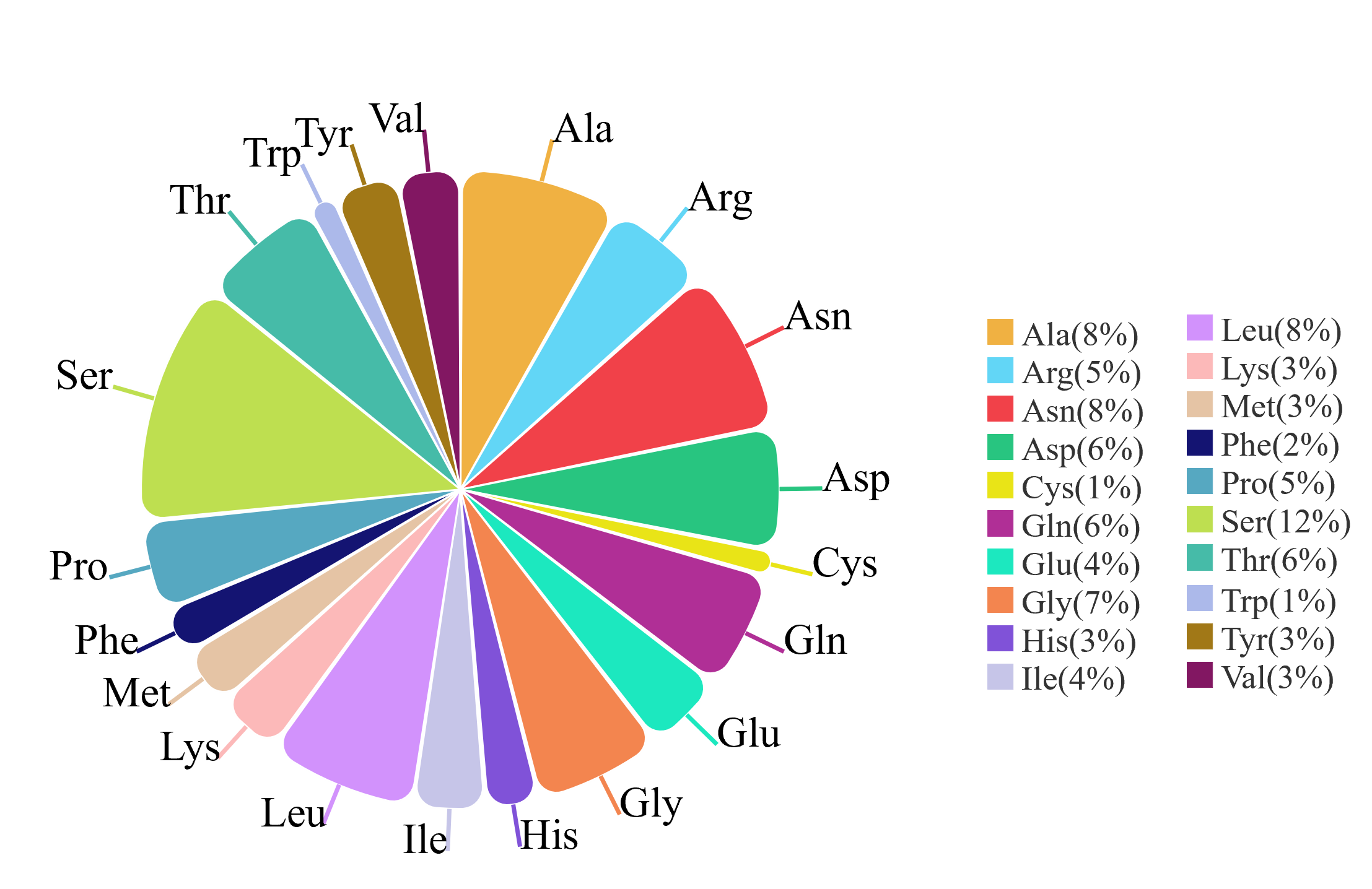

Supplement: Supplementary Figure 3 — The amino acid compositon and content of PmBBM protein. [file Image3.tif]

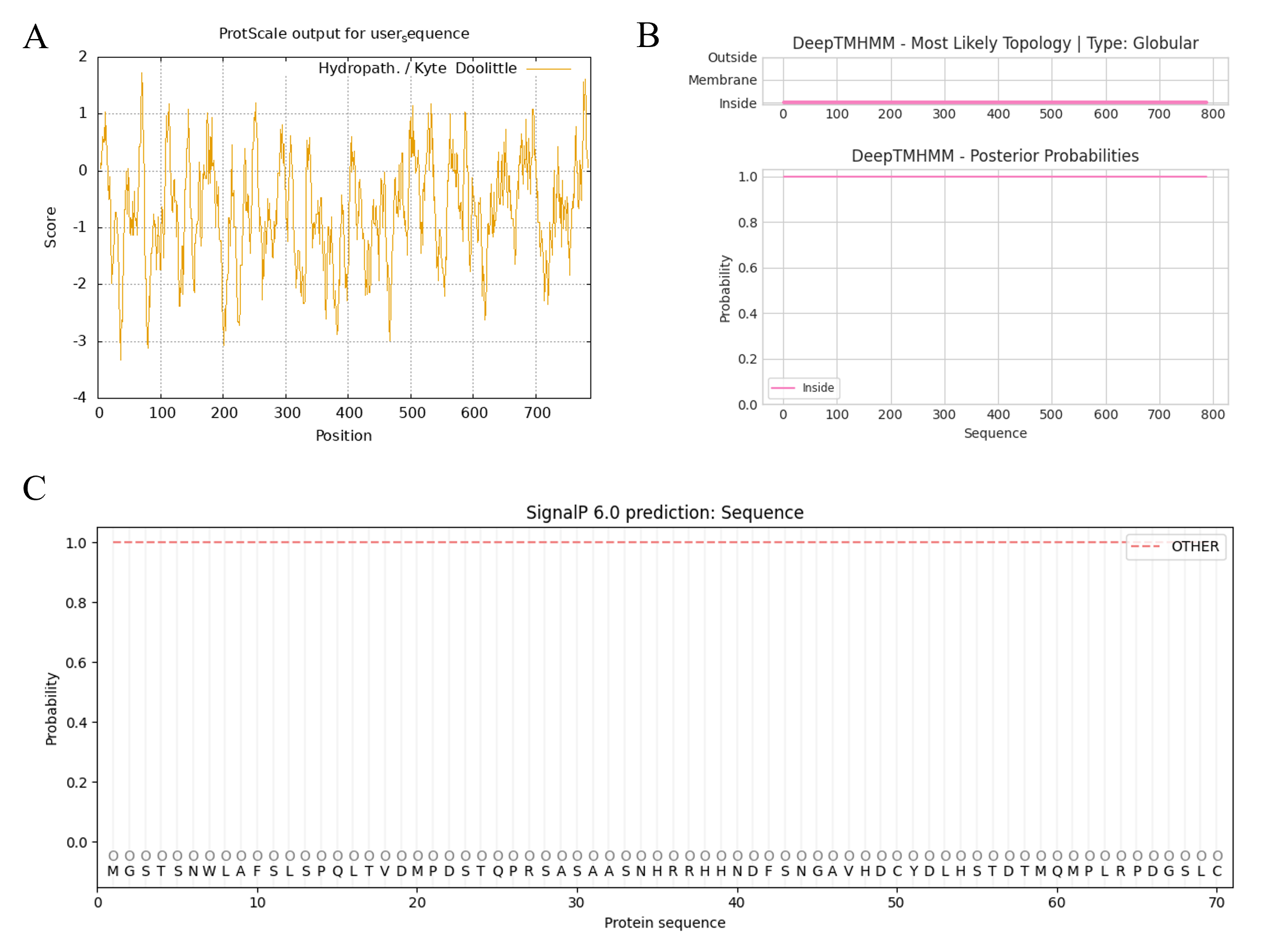

Supplement: Supplementary Figure 4 — Analysis of PmBBM protein. (A) Analysis of hydrophobicity/ hydrophilicity of the PmBBM protein. (B) Prediction of transmembtane structure of the protein. (C) Analysis of signal peptide of the PmBBM protein. [file Image4.tif]

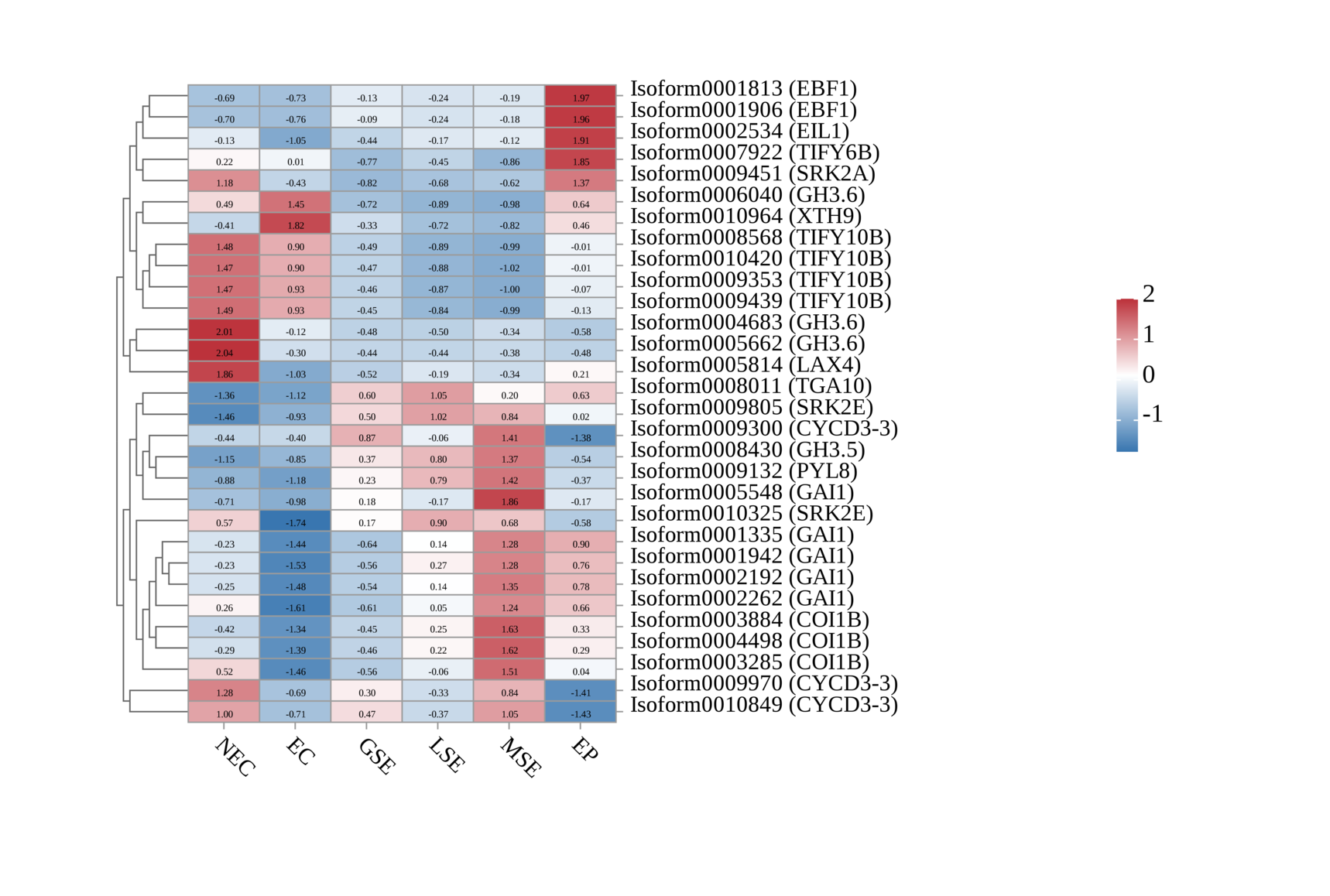

Supplement: Supplementary Figure 5 — Expression dynamics of plant hormone signal transduction genes during somatic embryogenesis. [file Image5.tif]
